# The Development and Validation of the Antisocial Preferences Scale

**DOI:** 10.3390/ijerph20032366

**Published:** 2023-01-29

**Authors:** Bartłomiej Skowroński

**Affiliations:** Institute of Social Prevention and Resocialization, Faculty of Applied Social Sciences and Resocialization, University of Warsaw, ul. Podchorążych 20, 00-721 Warsaw, Poland; b.skowronski@uw.edu.pl

**Keywords:** antisocial preferences, antisocial attitudes, antisocial preferences scale, attitudes

## Abstract

The aim of this study was to create a new instrument measuring antisocial preferences based on the Theory of Social Derailment of Czesław Czapów, who indicated the role of antisocial preferences in predicting antisocial behavior. The measures used were the Antisocial Preferences Scale (APS), BPAQ, Mach-IV, and IVE questionnaires. The participants were 718 prisoners. CFA techniques were used to investigate the construct validity of the Antisocial Preferences Scale. Four alternative models of the APS were specified and tested, namely: one-factor, second-order, multi-factor, and bi-factor. CFA analyses revealed that the best-fitting model was the bifactor. This conceptualization contains seven subscales, namely: aggressiveness, lack of guilt or remorse, breaking legal norms, incapacity for mutually intimate relationships, impulsiveness, risk-taking, and egocentrism.

## 1. Introduction

### 1.1. Antisocial Behavior

The term “antisocial behavior” is used as “a description for all behaviors, attitudes, and personality traits that people engage in that appear to be dysfunctional, in that they often have negative interpersonal and societal outcomes” [1]. Such behavior is not only of interest to social sciences, public policy, and public services but also poses a conceptual challenge [2]. As noted by dos Santos et al. [3], this type of behavior is variously defined and known under various terms: juvenile delinquency [4], antisocial personality disorder [5], conduct problems [6], disruptive behavior [7], and psychopathy [8,9,10].

Dos Santos et al. [3] observe that “despite the theoretical and methodological advances observed in the last few years, there is no consensus on the nature and dimensionality of antisocial behavior”. They explain: “Although these constructs may appear to be synonymous, most research in the area was carried out from a variety of conceptual perspectives and different approaches where the definition and the identification of the target participants were based on different criteria and methodological procedures, which makes the integration of the results problematic and suggests that the constructs are different” [3].

However, at least two perspectives can be highlighted. The first perspective is juridical, with the antisocial label applied to individuals who break the law [11]. Some authors give examples of antisocial behaviors, such as: shoplifting, bicycle theft, breaking into a house, using threats or force, bullying, fighting, and carrying a weapon [12,13,14,15].

The other perspective is related to classification systems such as the Diagnostic and Statistical Manual of Mental Disorders.

There are different approaches to the assessment of antisocial behavior, too. The first approach is associated with personality measurement (interviews, rating scales, self-report tests/personality inventories, e.g., the Jesness Inventory). Another one is the behavioral checklist, an example of which is the Psychopathy Checklist—Revised [16]. Finally, the third approach is DSM-oriented measures, an example of which is the Achenbach System of Empirically Based Assessment (ASEBA) [17], or the categorical classification. Focusing largely on the behavioral features while ignoring the underlying psychopathology, this approach has arbitrary cut-offs to classify the disorder. Moreover, some of the information obtained through a personality profile is lost in a categorical system [18].

A better understanding of what antisociality is and the development of new measures of antisociality have significant implications for prevention and intervention efforts.

### 1.2. Affective Component of Attitude

The classic model of attitude offered by Rosenberg and Hovland, known more widely as the multicomponent model, posits that attitude consists of affective, cognitive, and behavioral components [19,20]. Empirical studies have demonstrated that both the cognitive and affective components of attitude are associated with the overall attitude evaluation [21,22]. The components pointed out by Rosenberg and Hovland are also included in newer concepts [23]. Ajzen and Fishbein [24] recently postulated that, when measuring attitudes within the framework offered by the theory of reasoned action and the theory of planned behavior, one should take both cognitive and affective components into account. Attitude researchers exploring attitude–behavior relations are increasingly interested in the affective component of attitude [25,26,27]. A few recent studies have shown that in the case of some behaviors, affective attitudes are a stronger predictor of intentions and actions than cognitive attitudes [28].

There is no consensus on the definition of the affective/emotional component of attitude. Conner and colleagues [27] pointed out that affective attitude was conceptualized in different ways. According to these authors, the measures used in the research were “usually labeled anticipated affective reactions (AAR)” [27]. Moreover, affective assessment can be dichotomized in three ways: (1) affect that is expected to follow after the performance or nonperformance of a behavior vs. affect that is expected to occur while the behavior is being performed; (2) anticipated affective reactions, focused on self-conscious emotions (e.g., guilt), vs. affective attitudes, focused on hedonic emotions (e.g., excitement); (3) AAR have tended to examine the negative affect associated with the nonperformance of a behavior rather than the positive affect associated with the performance of a behavior [27]. Other authors, Breckler and Wiggins [29], define the affective aspect of attitude as the emotions and drives that are engendered by a specific attitude object.

A Polish scholar Czesław Czapów was probably one of the first researchers to indicate the role of emotional components in explaining antisocial behavior based on his concept of attitude being part of his Social Derailment Theory. Czapów believed that attitude was a mental system consisting of an external function and an internal one. According to him, activities are the external function, while motivation is the internal function [30]. Motivation comprises perceptive and preferential dispositions. The former is related to the cognitive belief system, while the latter is related to the emotional system [30]. Moreover, Czapów postulated that the affective component of attitudes was related to preferential dispositions (“I like seeing people fear me”) as opposed to cognitive dispositions (“There is nothing wrong with beating up a stealer”) [30].

### 1.3. Behavioral Prediction Based on Antisocial Attitudes

Antisocial attitude is a concept underlying antisocial behavior [23,30,31,32,33]. Antisocial attitudes are also one of the Central Eight risk/need factors in the Risk-Need-Responsivity Model [23]. There is strong evidence that antisocial attitudes, including emotional factors, are among the factors that predict criminal behavior [23,33,34,35,36,37,38,39].

Currently, two models of diagnosis dominate in psychology and psychiatry, namely categorical and dimensional. An example of the former is the Diagnostic and Statistical Manual of Mental Disorders (DSM), being a formal classification of mental health disorders and features symptoms (i.e., ASPD criteria). The example of the latter is a reliable and valid instrument measuring antisociality. Some authors argue that categorical assessment is associated with the loss of clinically relevant information [40]. There is strong evidence that dimensional models are more effective in terms of predictive validity [40,41,42,43].

### 1.4. The Present Study

Several instruments measuring criminal/antisocial attitudes are available in the literature, inter alia, the Psychological Inventory of Criminal Thinking Styles (PICTS) by Walters [44]; the Criminal Attribution Inventory (CRAI) by Kroner and Mills [32]; the Avoidance Responsibility Scale (ARS) by Powell, Rosen, and Huff [45]; The How I Think Questionnaire (HIT) [46]; The Pride Delinquency Scale (PID) [47]; The Criminal Sentiments Scale-Modified (CSS-M) [48]; The Measures of Criminal Attitudes and Associates (MCAA) [31]. The instruments listed above have limitations. Some of the scales of the Psychological Inventory of Criminal Thinking Styles (PICTS) are not reliable; this instrument is also not parsimonious. The next tool, namely the Criminal Attribution Inventory (CRAI), seems to be limited in its usefulness for predicting criminal behavior. In turn, the How I Think Questionnaire (HIT) is limited as it was designed for adolescents; the Avoidance Responsibility Scale (ARS) is limited in its usefulness for predicting antisocial behavior; it has not been found that the Pride Delinquency Scale (PID) predicts antisocial behavior.

The extent of antisocial content should be significantly different from non-offenders if attitudes have to be useful for the prediction of offending [49]. It seems that the DSM-V diagnostic criteria for Antisocial Personality Disorder (ASPD) may be taken into account as criteria of the new tool predicting antisocial behavior. There is strong evidence that ASPD predicts crime [50,51,52]. Moreover, people in correctional mental health settings have higher rates of antisocial personality disorder than people in the general community [53].

Antisocial attitude [30,54] comprises perceptive (cognitive belief system) and preferential dispositions (emotional system). While the Antisocial Beliefs Scale exists [55], there is a lack of a tool for measuring antisocial preferences.

Despite the growing number of instruments measuring antisocial attitudes, there is a lack of new tools assessing antisocial preferences and measuring the affective component of attitudes based on the antisocial personality disorder criteria. The goal of the study was to design a new instrument that would measure antisocial preferences. Additionally, there is a need to make new instruments measuring antisocial preferences, which could be used before and after intervention programs aimed at offenders. The next aim of the study was to develop and validate the construct validity and divergent criterion validity of a new measure of antisocial attitudes.

## 2. Materials and Methods

### 2.1. Sample and Procedure

A group of 800 individuals serving prison sentences and 339 non-offenders were approached. The non-offenders group was the only comparison group.

#### 2.1.1. Sample 1

The research was conducted in penal institutions administrated by the District Inspectorate of the Prison Service in Warsaw, Poland. Self-report questionnaires delivered to all prisons were anonymous. Before starting the research study, the prison staff was instructed about the procedure of the study. All questionnaires were distributed among prison inmates by the prison personnel, who also explained the nature of the study and provided a summary of the informed consent. This was necessary because prison inmates are a vulnerable population who might otherwise have felt compelled to take part in this study. Individuals were instructed to place completed questionnaires in unmarked envelopes and return them to the data collector. The prison staff monitored data collection, and the questionnaires were returned to the research team. 752 prison inmates returned completed questionnaires. Response rate was 89.75%. Due to significant missing data, some participants were excluded. Finally, data from 718 prisoners were included in the further analysis. 

The survey revealed that 32 prisoners (4.45%) had committed murder (Article 148 of the Penal Code), 147 (20.47%) were serving a sentence under Article 207 (mistreatment), and 66 (9.19%) were in prison for offenses under Article 286 (fraud, ransom). 

#### 2.1.2. Sample 2

Data were collected from non-offenders, too. Students of social prevention and resocialization distributed the questionnaires among adult men who were without a criminal record. In addition, in this case, informed consent was obtained from participants, and training for students was provided by the research team prior to data collection. As regards the control group, 400 individuals were approached; 366 of them returned completed surveys. Ultimately, 339 questionnaires were included in the analysis, as some of those returned were excluded due to missing data. The oldest and the youngest respondents were born in 1979 and 1998, respectively. The demographic characteristics are presented in Table 1 below.

### 2.2. Measures

Antisocial Preferences. To measure antisocial preferences, the Antisocial Preferences Scale (APS) was used. The APS is a 35-item self-report tool measuring antisocial preferences in forensic populations. It consists of seven subscales, namely: (1) Aggressiveness (AG), (2) Lack of Guilt or Remorse (LSGR), (3) Breaking Legal Norms (BLN), (4) Incapacity for Mutually Intimate Relationships (IMIR), (5) Impulsiveness (IMP), (6) Risk-Taking (RT), and (7) Egocentrism (EG). All items are rated on a 5-point Likert scale (1 = strongly disagree to 5 = strongly agree).

#### Development of the Antisocial Preferences Scale

The theoretical basis for the APS is Czesław Czapów’s concept of attitude [30]. A Polish scholar indicated the role of the emotional component in explaining antisocial behavior and focused on two elements: cognitive beliefs and emotional preferences. These components are related to behavior [30,54,56]. The APS consists of emotional preferences. The antisocial personality disorder (ASPD) criteria of the alternative model showed in the “Emerging Measures and Models” chapter of the DSM–5 manual, section III; [57] were taken into account in the development of the new instrument. Criterion A of the DSM–5 alternative model refers to impairment in self and interpersonal functioning (e.g., egocentricity and absence of internal prosocial standards and failure to conform to lawful behavior; lack of concern for others, lack of remorse, exploitativeness, use of deceit, coercion, dominance, and intimidation to fulfill interpersonal needs [57]. Criterion B for personality pathology refers to the presence of maladaptive personality traits, namely: manipulativeness, deceitfulness, callousness, and hostility (the antagonism) combined with irresponsibility, impulsivity, and risk-taking (the disinhibition) [57,58].

Based on the ASPD criteria, seven dimensions of antisocial preferences were extracted and discussed by a panel of 11 experts (specialists in the field of social rehabilitation). In the first stage of the construction of the measure, 140 items were extracted, each of them rated on a 5-point Likert scale (1 = strongly disagree to 5 = strongly agree). The procedure proposed by Lawshe was used to assess the content validity of the new measure [59]. The initial item pool was reduced to 35 items (five per dimension). The Cronbach’s alpha coefficients for the dimensions of APS are: (1) Aggressiveness = 0.81, (2) Lack of Guilt or Remorse = 0.76, (3) Breaking Legal Norms = 0.79, (4) Incapacity for Mutually Intimate Relationships = 0.82, (5) Impulsiveness = 0.79, (6) Risk Taking = 0.74, and (7) Egocentrism = 0.79. Moreover, McDonald’s omega coefficients for the subscales are very similar: 0.81 for Aggressiveness, 0.77 for Lack of Guilt or Remorse, 0.79 for Breaking Legal Norms, 0.81 for Incapacity for Mutually Intimate Relationships, 0.80 for Impulsiveness, 0.74 for Risk Taking, and 0.80 for Egocentrism. The Antisocial Preferences Scale had good internal reliability.

I predicted that the APS factors should correlate significantly with external variables: aggression [60], impulsiveness [61], and manipulativeness [62]. To test the correlations between the APS dimensions and the external variables listed above, three measures were used: the Buss-Perry Aggression Questionnaire (BPAQ) measuring aggression, the Mach-IV measuring Machiavellianism, and the IVE Questionnaire.

Aggression. To measure aggression, the Buss-Perry Aggression Questionnaire (BPAQ) was administered. The questionnaire consists of 29 items and measures four factors: physical aggression, verbal aggression, anger, and hostility. Its items are rated on a 5-point Likert scale (1 = extremely uncharacteristic, 2 = somewhat uncharacteristic, 3 = neither uncharacteristic nor characteristic, 4 = somewhat characteristic, 5 = extremely characteristic). Cronbach’s α coefficient for the whole scale was 0.80; as far as the subscales are concerned, it ranged from 0.77 for Hostility to 0.85 for Physical Aggression [63].

Machiavellianism. To measure Machiavellianism, the Mach-IV was used. The questionnaire measures three dimensions: (1) interpersonal tactics, (2) cynical views of human nature, and (3) utilitarian morality. The instrument consists of 20 self-report items, each of them scored on a 7-point Likert scale (1 = strong disagreement to 7 = strong agreement). Cronbach’s α for the whole scale was 0.73 [64].

Impulsivity. The IVE Questionnaire was used to measure the personality traits of impulsivity, venturesomeness, and empathy. This instrument consists of 63 items using a yes/no response format. Cronbach’s α coefficients are the following: for Impulsivity, 0.86 for women and 0.76 for men, for Venturesomeness 0.90 for women and 0.85 for men; and for Empathy, 0.77 for both genders [65].

### 2.3. Analytical Procedure

The four models were tested using confirmatory factor analysis (CFA): one latent factor model (M1), a multifactor model (M2), a second-order model (M3), and a bifactor model (M4). 

Kline recommends that at least four indicators should be used to test which model is the best fit for the data: (1) RMSEA (root mean square error of approximation) and (2) SRMR (standardized root mean square residual; [66,67], (3) TLI (Tucker–Lewis index) and (4) CFI (comparative fit index; [66,68]. RMSEA value should be close to 0.06 [69]. Kline [66] recommends that the CFI value should be higher than or equal to 0.90, and TLI should be higher than 0.95. Moreover, the cut-off for SRMR indicating a good fit is 0.08 [66].

All calculations were carried out using Mplus 8.2 [70]. Descriptive statistics, the Mann–Whitney U test, Pearson correlation coefficient, discrimination, and threshold parameters for the Graded Response Model were calculated using SPSS version 28. Additionally, the reliability of APS was provided by examining the composite reliability for a total score.

Graded Response Model (GRM) was used to examine the likelihood of responding in a particular response category. GRM is a type of polytomous IRT model [71]. It is an extension of the 2PL model for ordered scored items like a Likert-type rating.

## 3. Results

### 3.1. Descriptive Statistics

All descriptive statistics for the seven factors of the Antisocial Preferences Scale are provided in Table 2.

Descriptive statistics for BPAQ, MACH-IV, and IVE for the prisoners group are shown in Table 3.

### 3.2. Confirmatory Factor Analysis Results and Correlations between APS Dimensions

Table 4 presents the fit indices of all alternative models of the APS. The bifactor model has the best fit when compared with other estimated models; it showed slightly lower RMSEA and SRMR values and slightly higher TLI and CFI values than the remaining models. 

All of the standardized factor loadings for the general factor and the factors representing seven antisocial preferences are shown in Table 5 below.

The seven factors of the Antisocial Preferences Scale were found to be intercorrelated (see Table 6). Most of the correlations were moderate and positive. The correlation between risk-taking and incapacity for mutually intimate relationships was lowest and positive but above 0.40.

### 3.3. Criterion Validity

Table 7 presents the correlations between the seven APS factors and external variables. Aggressiveness (AG) correlated positively with the BPAQ total score, the remaining dimension of aggression, namely: physical, verbal aggression, anger and hostility (BPAQ), impulsiveness and venturesomeness (IVE), cynical views of human nature, interpersonal tactics, utilitarian morality (MACH-IV) and negatively with empathy (IVE). The correlation between the lack of guilt or remorse and empathy (IVE) was negative. On the other hand, the association between the lack of guilt or remorse and the remaining variables was positive. Breaking legal norms was a strong correlate of impulsivity. Moreover, impulsiveness correlated positively with all dimensions of the BPAQ, MACH-IV, and IVE. Risk-taking correlates strongly with impulsivity. Egocentrism and incapacity for mutually intimate relationships correlate negatively with empathy (IVE). The associations listed above were all expected.

### 3.4. Sample Comparisons

Table 8 presents the differences between prisoners and non-offenders (comparison group). All of them are statistically significant at *p* < 0.001. The mean scores of prisoners were significantly higher than the means of controls without a criminal record.

### 3.5. Composite Reliability

In order to assess the internal reliability of the Antisocial Preferences Scale, a composite reliability analysis was performed. The antisocial preferences total score demonstrated very good internal reliability (0.96).

### 3.6. Items Discrimination

Discrimination and the threshold parameters for each item of the Antisocial Preferences Scale are shown in Table 9. Item information curves for the all dimensions and Kernel Density estimation for ability estimates are available at Supplementary Materials.

In the first factor (aggressiveness), item discrimination varies between 1.902 (item_22) and 2.589 (item_1), and the average discrimination is 2.23. 

When the threshold parameters are examined, with a 50% probability, then the average ability level required for persons to mark categories higher than 1 instead of 1 is −0.440; the average ability level required to mark categories higher than 2 instead of 2 is 0.501; the average ability level required for to mark categories higher than 3 instead of 3 is 1.223, and finally, the average ability level required for individuals to mark categories for 5 instead of 4 is 1.847. In the second dimension (lack of guilt or remorse), item discrimination varies between 1.536 (item_30) and 2.166 (item_16), and the average discrimination is 1.959. 

In the next dimension (breaking legal norms), item discrimination varies between 1.230 (item_31) and 3.926 (item_3), and the average discrimination is 2.410. 

In turn, in the fourth dimension (incapacity for mutually intimate relationships item discrimination varies between 1.846 (item_11) and 2.873 (item_4), and the mean of discrimination is 2.293. 

In the fifth dimension, namely impulsiveness, item discrimination varies between 1.492 (item_5) and 2.756 (item_19), and the average discrimination is 2.214. 

In the next dimension (risk taking), item discrimination varies between 1.531 (item_20) and 2.271 (item_27). The average discrimination is 1.769.

Finally, in the last dimension (egocentrism), item discrimination varies between 1.754 (item_35) and 2.640 (item_21), and the average discrimination is 2.202.

To sum up, the average discrimination for all dimensions is above 1.7. According to Baker [72], when the value of the discrimination parameter is 1.7 or above, then it is classified as very high.

## 4. Discussion

The current study was carried out with the primary aim of introducing the Antisocial Preferences Scale and evaluating its dimensionality and construct validity. Moreover, this paper sought to determine its internal reliability.

The fit indices for all alternative measurement models for the Antisocial Preferences Scale are acceptable, and the differences between those indices are slight. However, the bi-factor model was indeed found to offer the best explanation of the data. The parameters of the bi-factor model show that the factor loadings are lower for grouping factors than for general factors. This suggests that the Antisocial Preferences Scale is not multidimensional but rather a unidimensional measure. Similar results were found in a recent study of antisocial beliefs in prison inmates [55].

There is no consensus on the construct of antisociality. Some authors suggest that antisociality is a syndrome of problem behavior best structured in terms of one factor [73,74]. Other authors believe that antisocial behavior has a multidimensional nature [3,75,76]. The results presented in this article provide empirical evidence on the nature of antisociality, which seems to be unidimensional. This seems to be in line with the social derailment theory. The author of this approach perceived antisocial preferences as unidimensional rather than multidimensional [30,54,56].

The results of the present study also indicate that antisociality, which has been conceptualized in various ways, can be defined as an attitudinal inclination and successfully measured as such. Moreover, the previously defined factorial structure of the Antisocial Preferences Scale, based on the DSM-5 Antisocial Personality Disorder Criteria, was confirmed. This means it is a new promising tool to assess one of the components of antisocial attitudes, namely the affective component.

Composite reliability was calculated for the antisocial preferences total score, which is very high. This indicates that all items constantly measure the same construct. The Antisocial Preferences Scale is a promising alternative to the existing scales assessing antisocial attitudes. It demonstrates very high internal reliability of the whole scale measured by the composite reliability coefficient [77,78] and good internal consistency of the seven dimensions (<0.7) of the instrument measured by Cronbach’s Alpha coefficient. 

The correlations between the latent factors were above 0.50, except for the association between risk-taking and incapacity for mutually intimate relationships (0.43). If the latent factors are highly intercorrelated, some researchers recommend tests to verify whether the dimensions correlate differentially with external criteria. [79,80].

Aggressiveness and lack of guilt or remorse correlated highly with each other. On the other hand, these factors correlated differently with BPAQ dimensions and empathy (IVE). Breaking legal norms and aggressiveness correlated differently with anger, physical aggression, and utilitarian morality too. The next two dimensions of the APS, namely lack of guilt or remorse and egocentrism, correlated differently with utilitarian morality and cynical views of human nature. Breaking legal norms and risk-taking correlated highly with each other but on the other hand, these variables correlated differently with empathy, interpersonal tactics, and utilitarian morality. The correlation between aggressiveness and risk-taking was high too, but the association between aggressiveness and empathy was negative and significant; in turn, the association between risk-taking and empathy was not statistically significant. Moreover, aggressiveness is highly correlated with impulsiveness (0.69). These variables correlated differently with anger, verbal aggression, and similar construct measured by IVE (impulsivity). Aggressive and impulsive preferences are correlates of aggression measured by the BPAQ. This seems to be consistent with the conclusion that aggression is a frequent manifestation of antisocial personality disorder [61]. 

The differences between the group of prisoners and the group of men without a criminal record are statistically significant in the case of all variables measured by the Antisocial Preferences Scale. The means were significantly higher for prisoners than for the control group.

This study has important practical implications. The social rehabilitation process has been a challenge for social scientists [23]. The Antisocial Preferences Scale can be used in testing antisocial preferences among adults representing forensic populations, both before and after intervention programs (pretest vs. posttest). The theoretical underpinnings of the tool are the antisocial personality disorder criteria of the DSM-5 alternative model [81]. Persons with ASPD were found to be more prone to recidivism [82]. However, the recidivism-predicting power of the APS is an issue for future research to investigate.

The use of a self-report measure of antisocial preferences in various populations (including nonincarcerated samples) might allow for a more effective exploration of the relationship between criminal behavior and antisocial attitudes.

Future research should investigate the relationship of the Antisocial Preferences Scale to other established measures of antisocial behavior and antisocial attitudes in order to further elucidate its criterion validity. If this measure is found to map well onto antisociality, it might be useful in reducing the time of measuring antisociality in forensic and correctional samples. Moreover, future research should extend and replicate the findings of the present study in broader community-based samples and in any other setting where the assessment of antisociality can be conducted (e.g., forensic inpatient psychiatric settings).

The study has several methodological limitations. The analysis was based on data from a Polish prison population only. Different linguistic and cultural contexts should be included in future studies. Another limitation of the project was its cross-sectional design, which made it impossible to draw conclusions about the causal relationships between the factors of the Antisocial Preferences Scale and external criteria.

According to Hare and Neumann [83], the latest generation of risk assessment instruments has largely dispelled the belief that useful predictions cannot be made about antisocial behavior. Testing the predictive value of the Antisocial Preferences Scale is a task for future research.

## 5. Conclusions

The presented instrument can be used among individuals with a criminal history. Additionally, this instrument measuring antisocial preferences may be used before and after intervention programs aimed at offenders.

## Figures and Tables

**Table 1:** Descriptive statistics for demographic variables.

		Prisoners (*N* = 718)	Non-Offenders (*N* = 339)
Age in years (*M*/*SD*)		37/10.8	28.6/5.4
Residence (*N*/%)			
	Village	119/16.6	175/51.6
	Town (≤50,000)	167/23.3	118/34.8
	City (50,000–150,000)	106/14.8	9/2.7
	City (>150,000)	311/43.3	37/10.9
	Total	703/97.9	339/100
	No data	15/2.1	0
Education level (*N*/%)			
	Elementary	166/23.1	0
	Middle school	88/12.3	0
	Basic vocational	195/27.2	0
	Secondary vocational	114/15.9	38/11.2
	General Secondary	90/12.5	197/58.1
	Bachelor’s degree	25/3.5	36/10.6
	Master’s degree	25/3.5	68/20.1
	Total	703/97.9	339/100
	No data	15/2.1	0
Number of sentences (*N*/%)			
	1–5	480/66.85	
	6–10	155/21.58	
	11–15	19/2.64	
	16–20	12/1.67	
	20<	10/1.39	
	Total	676/94.15	
	No data	42/5.85	

**Table 2:** Descriptive statistics for APS factors: prisoners (*N* = 718); non-offenders (*N* = 339).

		Prisoners						Non-Offenders					
	Variables	*M*	*SD*	Skewness	Kurtosis	Min.	Max.	*M*	*SD*	Skewness	Kurtosis	Min.	Max.
1.	Aggressiveness	11.09	4.54	0.348	−0.728	5	24	7.33	1.86	0.626	−0.766	5	11
2.	Lack of guilt or remorse	11.74	4.24	0.120	−0.655	5	23	8.47	2.35	0.486	0.128	5	15
3.	Breaking legal norms	11.15	4.39	0.301	−0.698	5	25	6.98	1.64	0.340	−1.096	5	10
4.	Incapacity for mutually intimate relationships	9.80	4.26	0.597	−0.583	5	24	7.01	1.67	0.120	−1.388	5	10
5.	Impulsiveness	11.78	4.44	0.213	−0.586	5	25	8.38	2.17	0.210	−1.107	5	12
6.	Risk-taking	12.44	4.20	−0.101	−0.638	5	24	8.36	1.74	0.079	−0.967	5	12
7.	Egocentrism	11.33	4.33	0.269	−0.573	5	25	10.42	3.89	0.241	−0.802	5	19
8.	APS total score	79.35	25.27	0.08	−0.550	35	159	56.77	12.17	0.30	−1.20	38	78

**Table 3:** Descriptive statistics for BPAQ, MACH-IV, and IVE for the prisoners group.

	Variables	*M*	*SD*	Min.	Max.	Skewness	Kurtosis
BPAQ	Anger	18.82	5.29	7	33	0.228	−0.460
	Hostility	22.35	6.38	8	40	0.049	−0.191
	Physical aggression	22	6.79	9	45	0.391	−0.230
	Verbal aggression	15.04	3.80	5	25	−0.034	−0.034
	BPQA global score	78.22	18.97	35	136	0.188	0.188
MACH-IV	Interpersonal tactics	31.93	7.15	9	52	−0.101	0.023
	Cynical views of human nature	34.31	6.32	9	57	−0.336	1.474
	Utilitarian morality	7.98	2.57	2	14	−0.172	0.626
IVE	Impulsivity	9.72	4.56	1	19	0.041	−0.870
	Venturesomeness	9.57	2.85	2	16	−0.168	−0.567
	Empathy	10.34	2.65	3	19	0.302	0.087

Note: BPAQ—The Buss–Perry Aggression Questionnaire; MACH-IV—Machiavellianism Test; IVE—Eysenck’s Impulsivity Inventory.

**Table 4:** Fit indices for alternative measurement models for the Antisocial Preferences Scale.

Model	Test χ^2^ (*df*)	CFI	TLI	SRMR	RMSEA	RMSEA CI 90%
M1: ONE-FACTOR	3196.154 (560) *	0.913	0.908	0.056	0.081	[0.078, 0.084]
M2: MULTIFACTOR	2395.947 (553) *	0.939	0.935	0.048	0.068	[0.065, 0.071]
M3: SECOND-ORDER	2268.087 (539) *	0.943	0.937	0.045	0.067	[0.064, 0.070]
M4: BIFACTOR	2126.687 (525) *	0.947	0.940	0.045	0.065	[0.062, 0.068]

Note: * *p* < 0.001.

**Table 5:** Standardized factor loadings for the general factor and the factors representing seven antisocial preferences.

	Original Item Numbers	AG	LSGR	BLN	IMIR	IMP	RT	EG	General Factor
(1)	I often get into fights when someone irritates me.	0.091 *							0.764 **
(2)	I don’t like people who cry.		0.106 *						0.724 **
(3)	I could steal a petty thing only to experience a thrill of excitement.			0.449 **					0.743 **
(4)	I treat relationships with women as an unwanted obligation.				0.411 **				0.732 **
(5)	I am annoyed by people looking at me on the bus.					0.077 *			0.641 **
(6)	I am capable of doing many crazy things if someone persuades me to.						0.379 **		0.602 **
(7)	There is no place for sentiment in life: I only do what benefits me.							0.519 **	0.637 **
(8)	I like to see people fear me.	0.271 **							0.717 **
(9)	I don’t care if someone feels bad because of my behavior.		0.336 **						0.662 **
(10)	I could steal only the smallest things for fun.			0.432 **					0.724 **
(11)	I have never been in love with a woman.				0.306 **				0.656 **
(12)	Sometimes I flare up over nothing for no reason (at all).					0.377 **			0.692 **
(13)	I avoid engaging in something that does not give me “a thrill of excitement”.						0.068 *		0.585 **
(14)	I am only guided by my own good.							0.267 **	0.660 **
(15)	I sometimes humiliate others.	0.247 **							0.698 **
(16)	I am not in the habit of apologizing.		0.703 **						0.676 **
(17)	I could commit theft to experience a surge of adrenaline (“a thrill of excitement”).			0.392 **					0.677 **
(18)	I do not enter into relationships with women because they demand too much.				0.545 **				0.603 **
(19)	I very easily lose my temper.					0.521 **			0.655 **
(20)	I need constant changes in my life.						0.277 **		0.548 **
(21)	I try to care mostly for myself.							0.362 **	0.690 **
(22)	I wouldn’t be a real man if I didn’t want to fight others.	0.073 *							0.713 **
(23)	I do not inquire into whether I am doing the right thing.		0.081 *						0.706 **
(24)	I do not treat agreements with other people as something that must always be fulfilled.			0.117 **					0.675 **
(25)	I avoid entering into relationships with women because of the inevitable problems.				0.457 **				0.608 **
(26)	I quickly lose my patience when someone criticizes me.					0.267 **			0.626 **
(27)	I often feel the need for thrills.						0.519**		0.589 **
(28)	I don’t care much about other people’s fate.							0.277 **	0.642 **
(29)	I am excited by fist fighting.	0.074 *							0.715 **
(30)	There are times when I remain indifferent to the pain and suffering of others.		0.178 **						0.590 **
(31)	I only keep written agreements.			0.152 **					0.667 **
(32)	I avoid intimate relationships with women.				0.490 **				0.560 **
(33)	I am quarrelsome.					0.304 **			0.687 **
(34)	Being in danger is also exciting.						0.257 **		0.673 **
(35)	I rarely help other people.							0.164 **	0.676 **

Note: AG = aggressiveness; LSGR = lack of guilt or remorse; BLN = breaking legal norms; IMIR = incapacity for mutually intimate relationships; IMP = impulsiveness; RT = risk-taking; EG = egocentrism. Factor loadings are statistically significant at * *p* < 0.05 ** *p* < 0.001.

**Table 6:** Correlations between APS factors.

Factor	AG	LSGR	BLN	IMIR	IMP	RT	EG	APTS
Aggressiveness (AG)	1							
Lack of guilt or remorse (LSGR)	0.728 **	1						
Breaking legal norms (BLN)	0.702 **	0.666 **	1					
Incapacity for mutually intimate relationships (IMIR)	0.614 **	0.621 **	0.627 **	1				
Impulsiveness (IMP)	0.699 **	0.645 **	0.639 **	0.580 **	1			
Risk-taking (RT)	0.684 **	0.627 **	0.702 **	0.435 **	0.628 **	1		
Egocentrism (EG)	0.638 **	0.709 **	0.663 **	0.582 **	0.640 **	0.572 **	1	
Antisocial preferences total score (APTS)	0.873 **	0.858 **	0.860 **	0.766 **	0.832 **	0.798 **	0.826 **	1

Note: ** *p* < 0.001.

**Table 7:** Associations between the seven APS factors and external variables.

		Aggressiveness	Lack of Guilt or Remorse	Breaking Legal Norms	Incapacity for Mutually Intimate Relationships	Impulsiveness	Risk-Taking	Egocentrism	Antisocial Preferences Total Score
BPQA	Anger	0.334 **	0.265 **	0.263 **	0.145 **	0.449 **	0.271 **	0.299 **	0.350 **
	Hostility	0.318 **	0.278 **	0.293 **	0.162 **	0.386 **	0.307 **	0.285 **	0.350 **
	Physical aggression	0.480 **	0.387 **	0.375 **	0.253 **	0.490 **	0.349 **	0.381 **	0.469 **
	Verbal aggression	0.262 **	0.193 **	0.227 **	n.s.	0.311 **	0.237 **	0.228 **	0.258 **
	BPQA: total score	0.425 **	0.345 **	0.352 **	0.193 **	0.493 **	0.351 **	0.362 **	0.435 **
MACH-IV	Interpersonal tactics	0.284 **	0.278 **	0.311 **	0.199 **	0.240 **	0.265 **	0.294 **	0.322 **
	Cynical views of human nature	0.156 **	0.121 *	0.168 **	0.109 *	0.207 **	0.120 *	0.232 **	0.192 **
	Utilitarian morality	0.148 **	0.123 *	0.218 **	n.s.	0.185 **	0.167 **	0.224 **	0.193 **
IVE	Impulsivity	0.431 **	0.362 **	0.402 **	0.219 **	0.514 **	0.446 **	0.404 **	0.479 **
	Venturesomeness	0.265 **	0.224 **	0.217 **	n.s.	0.227 **	0.329 **	0.212 **	0.259 **
	Empathy	−0.182 **	−0.280 **	−0.141 **	−0.268 **	−0.102 *	n.s.	−0.295 **	−0.225 **

Note: AG = aggressiveness; LSGR = lack of guilt or remorse; BLN = breaking legal norms; IMIR = incapacity for mutually intimate relationships; IMP = impulsiveness; RT = risk-taking; EG = egocentrism. * *p* < 0.01. ** *p* < 0.001.

**Table 8:** Differences between prisoners and non-offenders on the dimensions of the Antisocial Preferences Scale.

	*M*_rank_		*U*	*Z*
	Prisoners	Non-Offenders		
Aggressiveness	608.26	361.12	64,791	−12.327 *
Lack of guilt or remorse	609.47	358.57	63,925.5	−12.531 *
Breaking legal norms	624.29	327.18	53,282.5	−14.853 *
Incapacity for mutually intimate relationships	589.73	400.38	78,097.5	−9.512 *
Impulsiveness	607.13	363.53	65,607	−12.151 *
Risk-taking	627.80	319.74	50,762	−15.363 *
Egocentrism	552.16	479.95	105,072	−3.603 *
Antisocial preferences total score	619.85	336.58	56,471.5	−14.083 *

Note: * *p* < 0.001.

**Table 9:** Discrimination and threshold parameters for the Graded Response Model.

Dimensions	Items	Item Parameters				
		a	b1	b2	b3	b4
Aggressiveness	Item_1	2.589	−0.364	0.571	1.233	1.900
	Item_8	2.350	−0.320	0.534	1.304	1.867
	Item_15	2.324	−0.499	0.468	1.121	1.782
	Item_22	1.902	−0.711	0.448	1.296	1.884
	Item_29	2.000	−0.306	0.486	1.163	1.805
	Mean	2.233	−0.440	0.501	1.223	1.847
Lack of guilt or remorse	Item_2	2.130	−0.686	0.405	1.311	2.076
	Item_9	2.042	−0.646	0.500	1.336	2.074
	Item_16	2.166	−0.500	0.554	1.234	1.870
	Item_23	1.925	−0.848	0.290	1.136	1.887
	Item_30	1.536	−0.874	0.385	1.156	2.202
	Mean	1.959	−0.710	0.426	1.234	2.021
Breaking legal norms	Item_3	3.926	−0.290	0.379	0.980	1.509
	Item_10	3.207	−0.302	0.518	1.136	1.713
	Item_17	2.387	−0.402	0.447	1.047	1.803
	Item_24	1.300	−0.794	0.603	1.644	2.946
	Item_31	1.230	−0.752	0.658	1.783	2.797
	Mean	2.410	−0.508	0.521	1.318	2.153
Incapacity for mutually intimate relationships	Item_4	2.873	0.023	0.817	1.656	2.337
	Item_11	1.846	0.212	0.881	1.700	2.466
	Item_18	2.626	−0.146	0.875	1.695	2.405
	Item_25	2.125	−0.320	0.757	1.593	2.261
	Item_32	1.998	0.036	0.886	1.545	2.249
	Mean	2.293	−0.039	0.843	1.637	2.343
Impulsiveness	Item_5	1.492	−0.775	0.375	1.336	2.243
	Item_12	2.595	−0.605	0.380	0.947	1.604
	Item_19	2.756	−0.431	0.509	1.145	1.877
	Item_26	1.910	−0.982	0.255	1.106	1.930
	Item_33	2.317	−0.523	0.521	1.224	2.066
	Mean	2.214	−0.663	0.408	1.151	1.944
Risk-taking	Item_6	1.854	−0.794	0.345	1.019	2.033
	Item_13	1.176	−0.977	0.392	1.779	2.939
	Item_20	1.531	−1.108	0.167	1.202	2.249
	Item_27	2.271	−0.847	0.136	0.999	1.654
	Item_34	2.015	−0.799	0.139	0.953	1.901
	Mean	1.769	−0.905	0.235	1.190	2.155
Egocentrism	Item_7	2.605	−0.579	0.476	1.221	1.670
	Item_14	2.054	−0.710	0.463	1.237	2.039
	Item_21	2.640	−0.533	0.560	1.084	1.887
	Item_28	1.960	−0.603	0.496	1.311	2.126
	Item_35	1.754	−0.675	0.590	1.327	2.040
	Mean	2.202	−0.620	0.517	1.236	1.952

## Data Availability

The data presented in this study are available on request from the corresponding author. The data are not publicly available due to privacy.

## Supplementary Materials

The following supporting information can be downloaded at: https://www.mdpi.com/article/10.3390/ijerph20032366/s1.
