# Supplementary material for: The Development and Validation of the Antisocial Preferences Scale
Source: Int J Environ Res Public Health. 2023 Jan 29;20(3):2366. doi: 10.3390/ijerph20032366 (PMC9916389; doi:10.3390/ijerph20032366)
Supplement: Supplementary file 1 [file ijerph-20-02366-s001.zip › ijerph-2134062-supplementary.pdf]

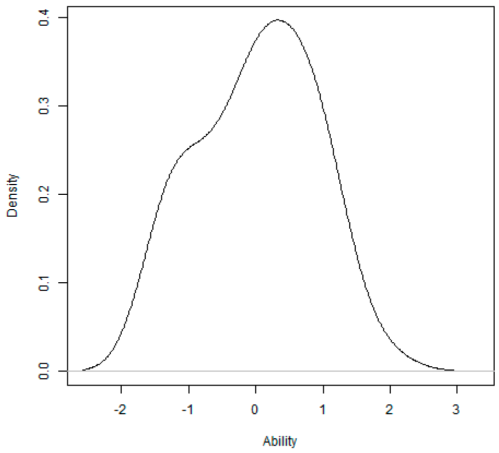

Figure S1. Kernel Density estimation for ability estimates (aggressiveness)

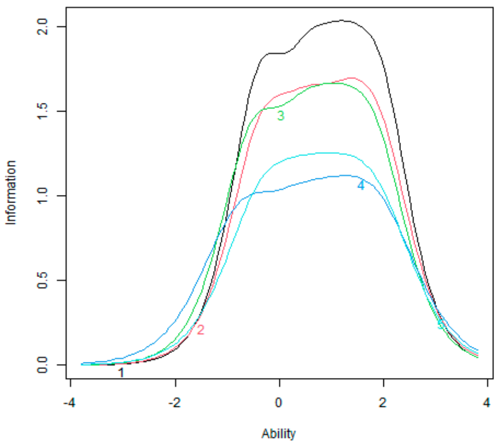

Figure S2. Item information curves for the aggressiveness dimension

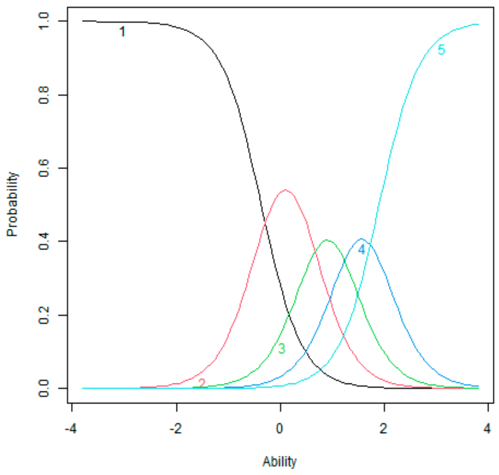

Figure S3. Item response category characteristic for item_1

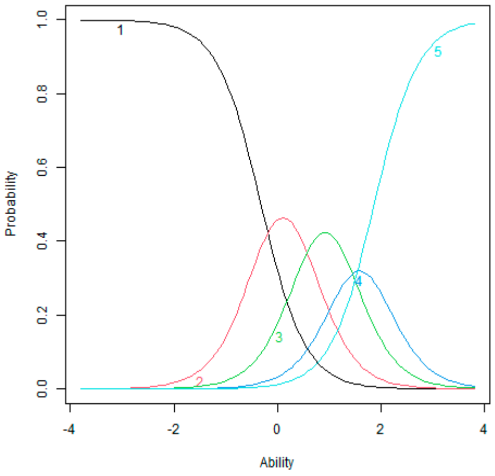

Figure S4. Item response category characteristic for item_8

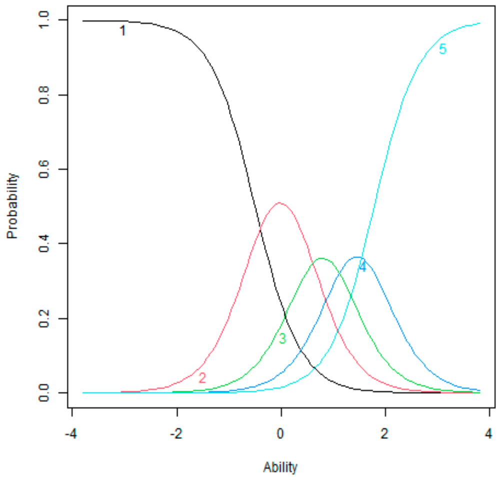

Figure S5. Item response category characteristic for item_15

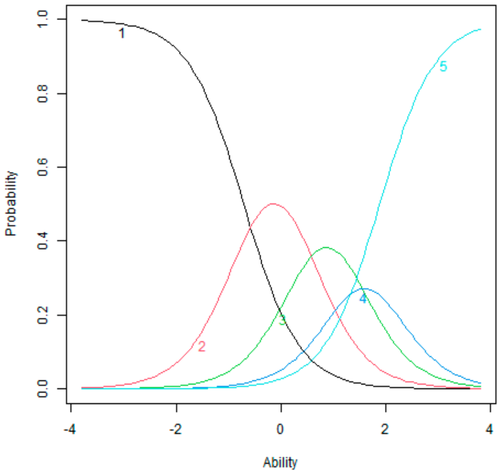

Figure S6. Item response category characteristic for item_22

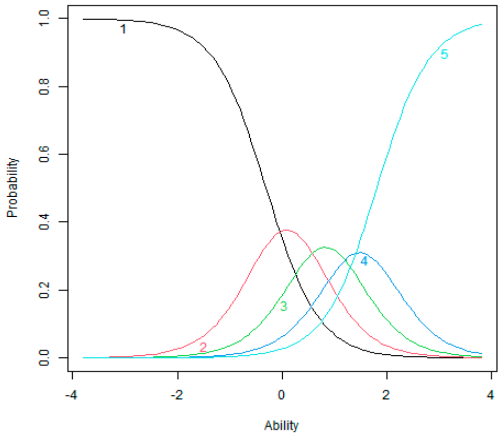

Figure S7. Item response category characteristic for item_29

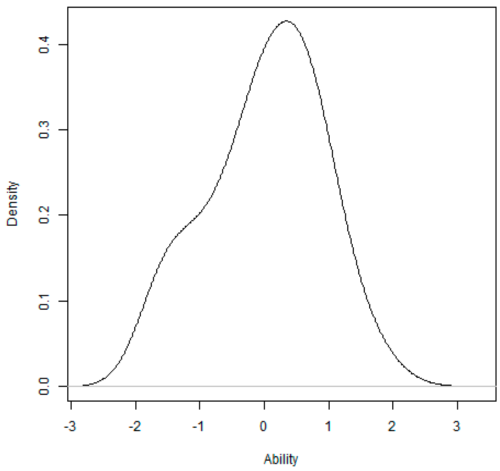

Figure S8. Kernel Density estimation for ability estimates (lack of guilt or remorse dimension)

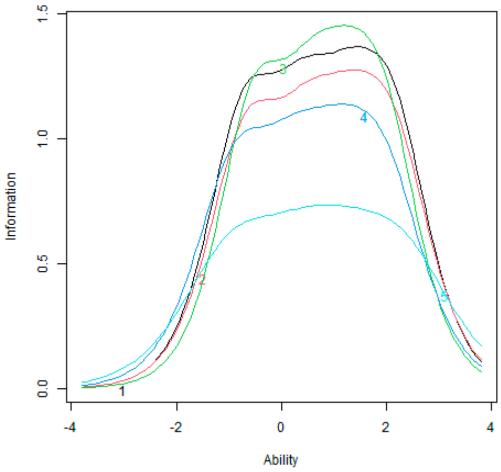

Figure S9. Item information curves for the lack of guilt or remorse dimension

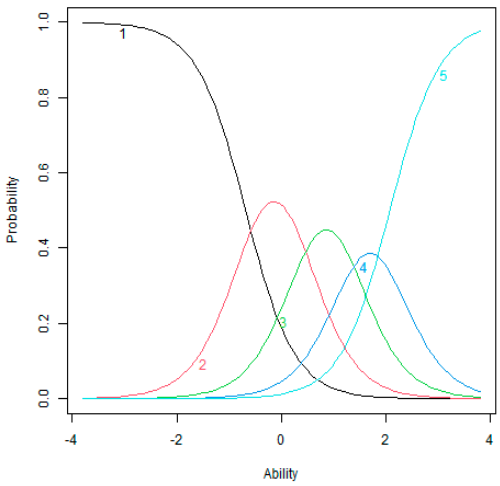

Figure S10. Item response category characteristic for item_2

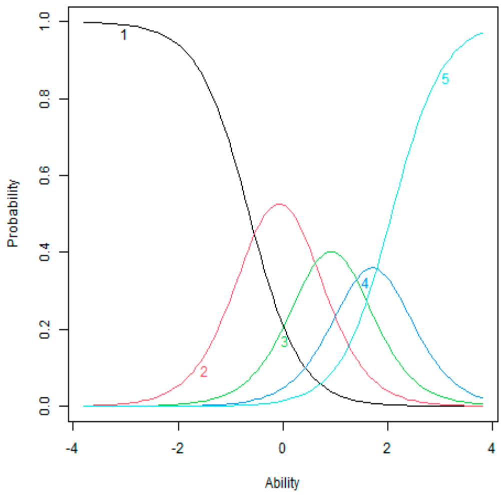

Figure S11. Item response category characteristic for item_9

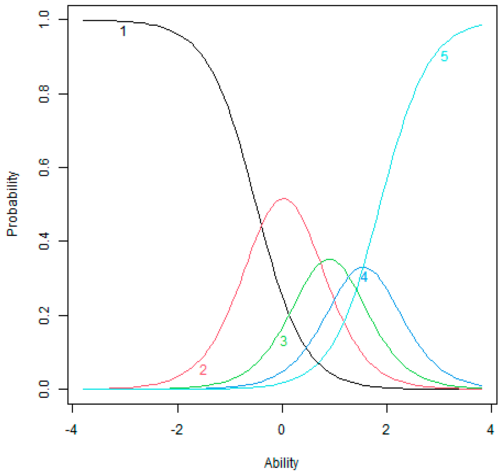

Figure S12. Item response category characteristic for item_16

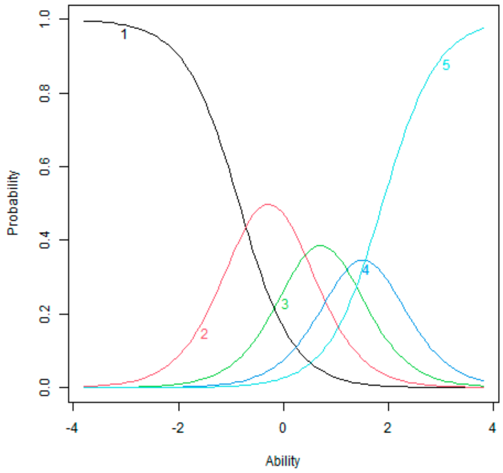

Figure S13. Item response category characteristic for item_23

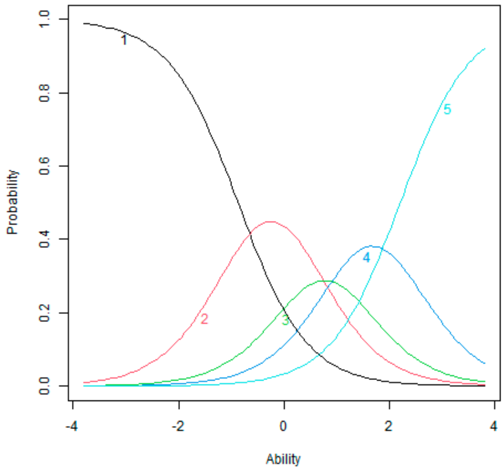

Figure S14. Item response category characteristic for item_30

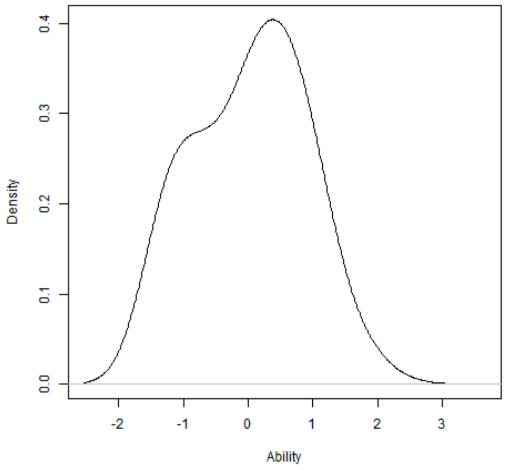

Figure S15. Kernel Density estimation for ability estimates (breaking legal norms)

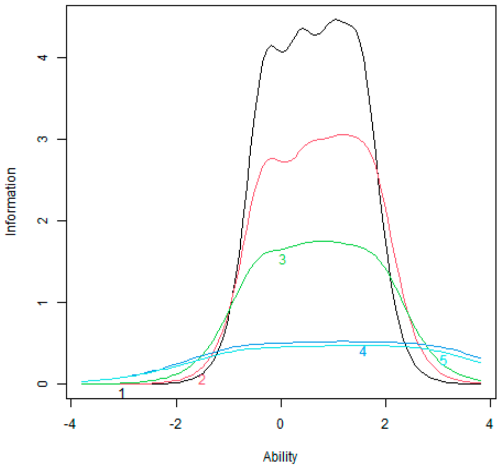

Figure S16. Item information curves for the breaking legal norms dimension

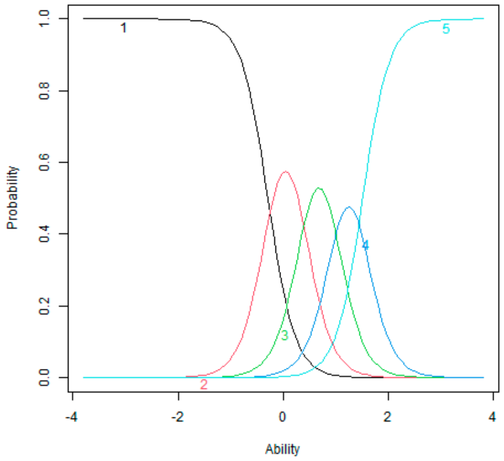

Figure S17. Item response category characteristic for item_3

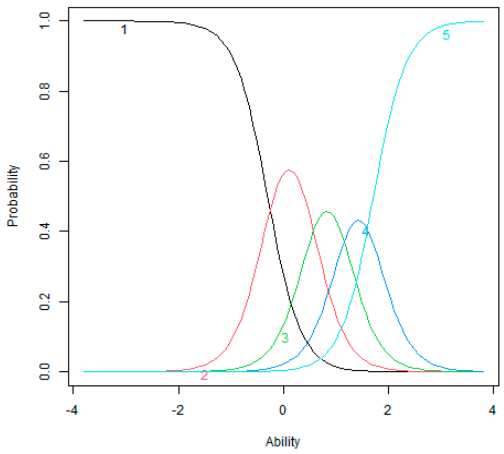

Figure S18. Item response category characteristic for item_10

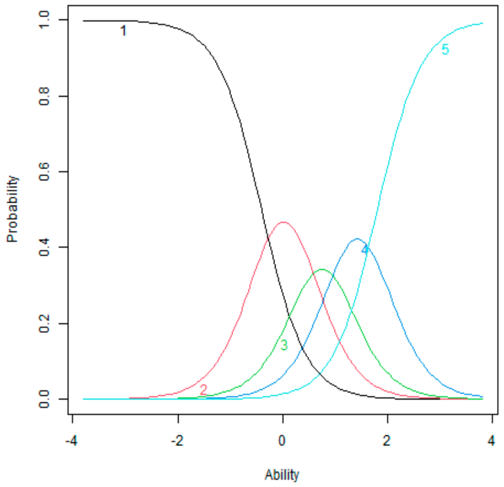

Figure S19. Item response category characteristic for item_17

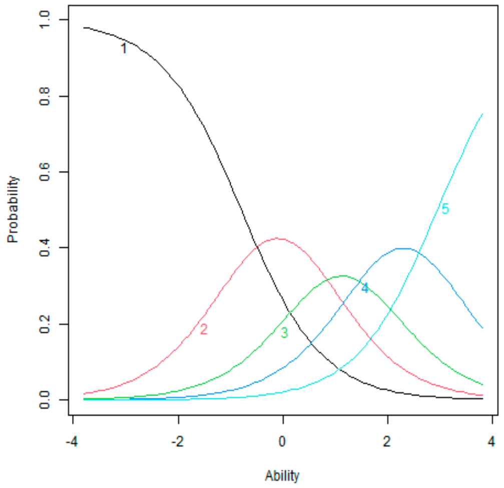

Figure S20. Item response category characteristic for item_24

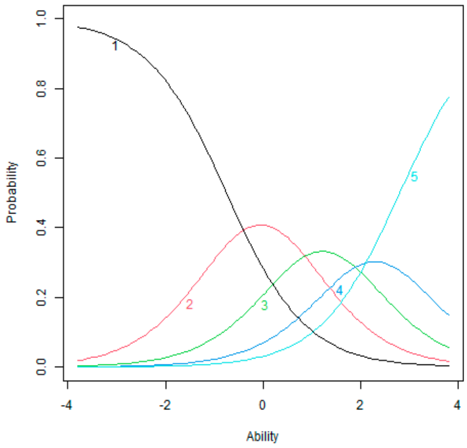

Figure S21. Item response category characteristic for item_31

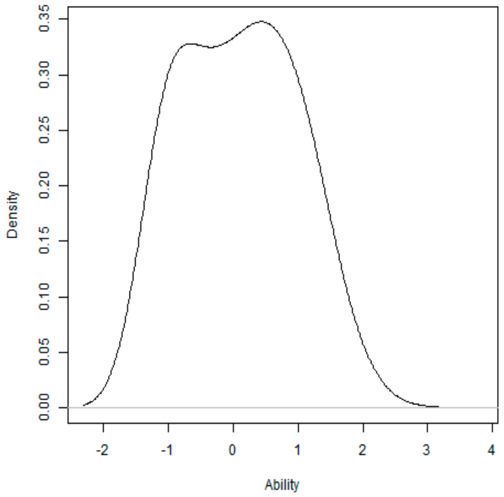

Figure S22. Kernel Density estimation for ability estimates (incapacity for mutually intimate relationships)

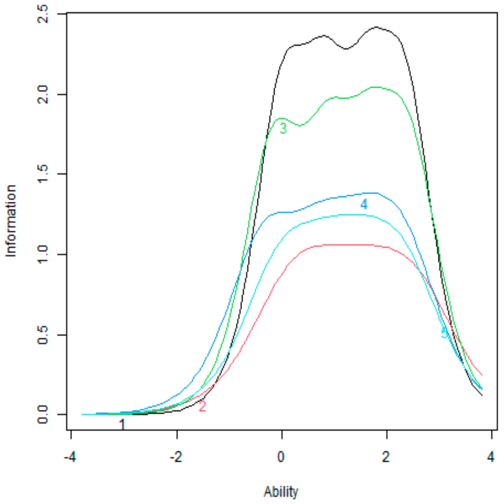

Figure S23. Item information curves for the incapacity for mutually intimate relationships dimension

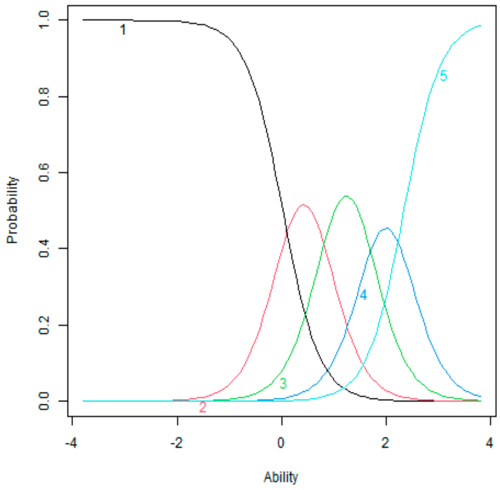

Figure S24. Item response category characteristic for item_4

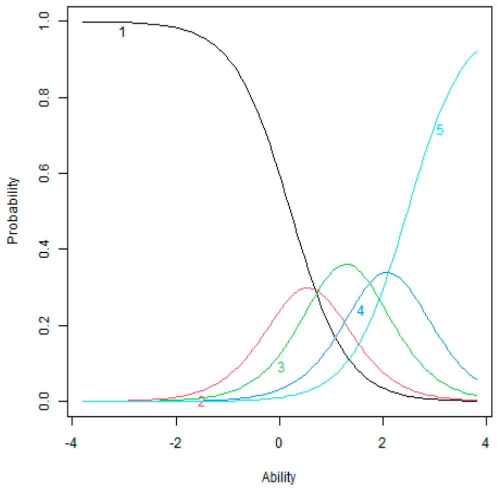

Figure S25. Item response category characteristic for item_11

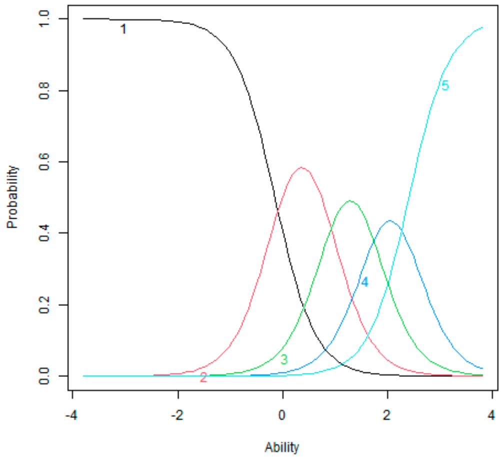

Figure S26. Item response category characteristic for item_18

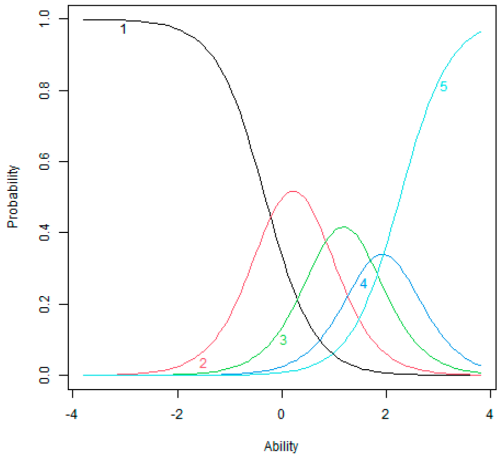

Figure S27. Item response category characteristic for item_25

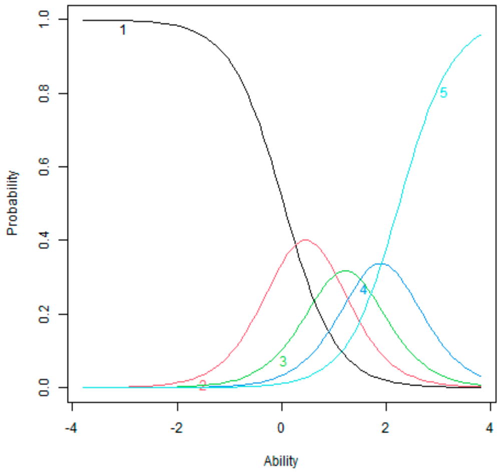

Figure S28. Item response category characteristic for item_32

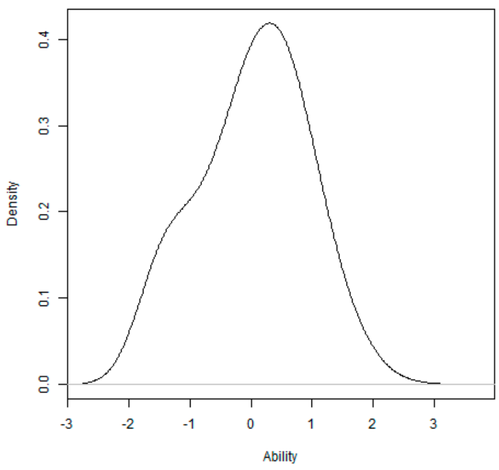

Figure S29. Kernel Density estimation for ability estimates (impulsiveness)

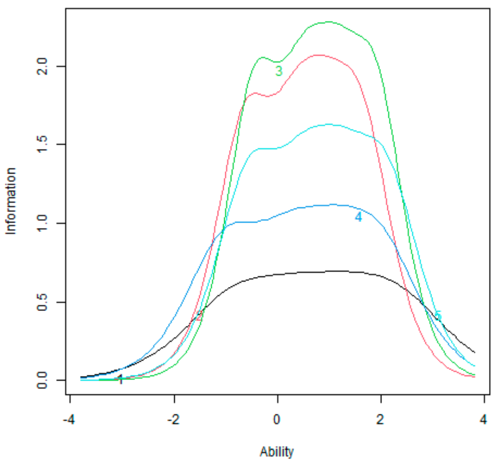

Figure S30. Item information curves for the impulsiveness dimension

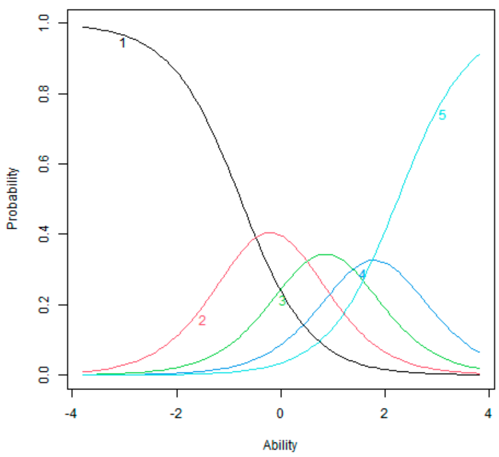

Figure S31. Item response category characteristic for item_5

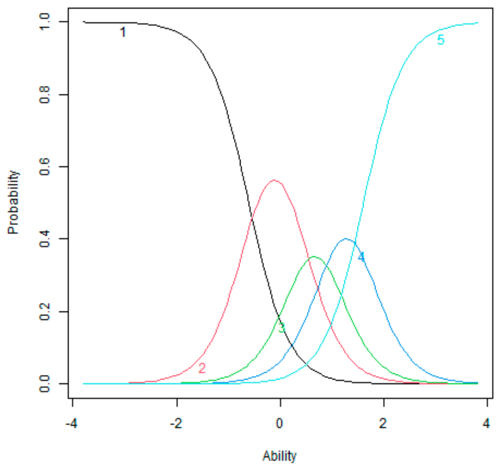

Figure S32. Item response category characteristic for item_12

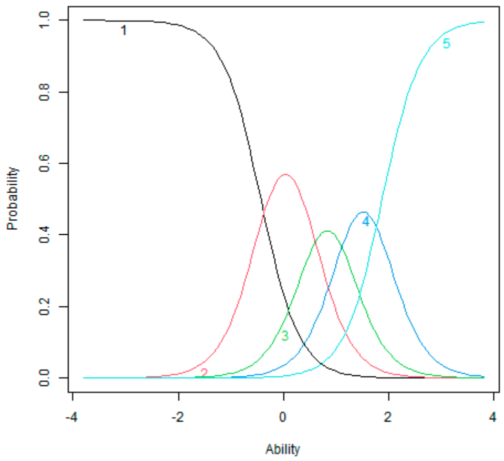

Figure S33. Item response category characteristic for item_19

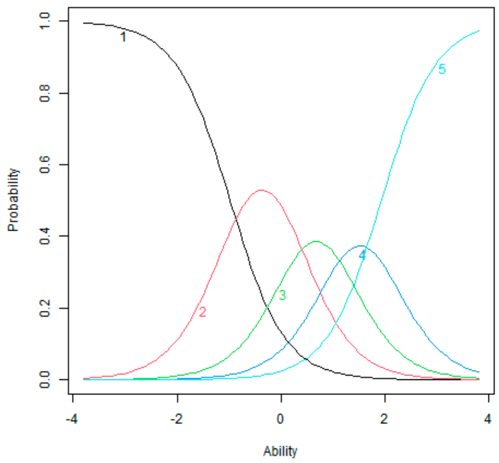

Figure S34. Item response category characteristic for item_26

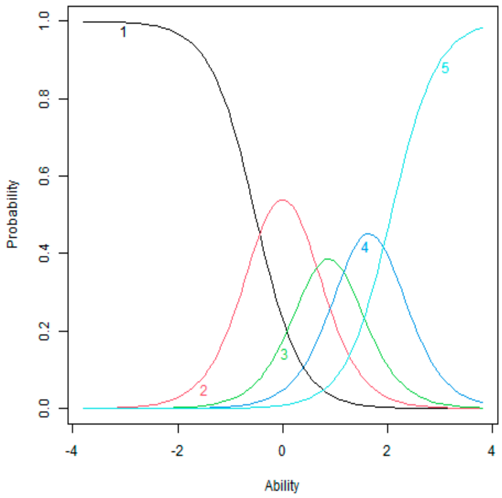

Figure S35. Item response category characteristic for item_33

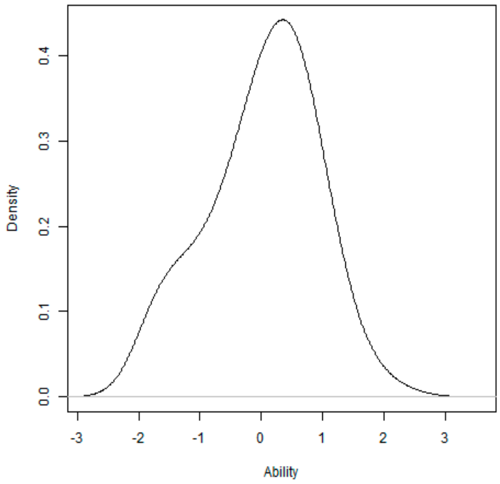

Figure S36. Kernel Density estimation for ability estimates (risk taking)

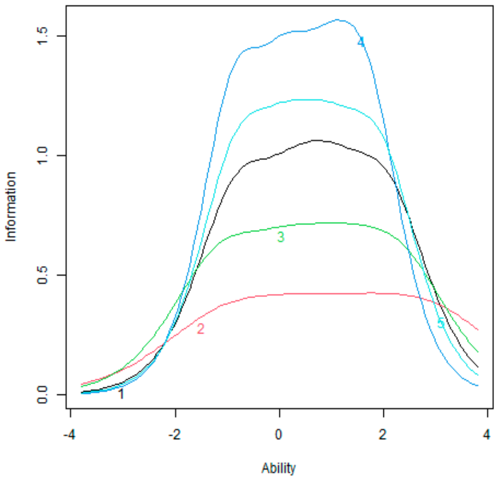

Figure S37. Item information curves for the risk taking dimension

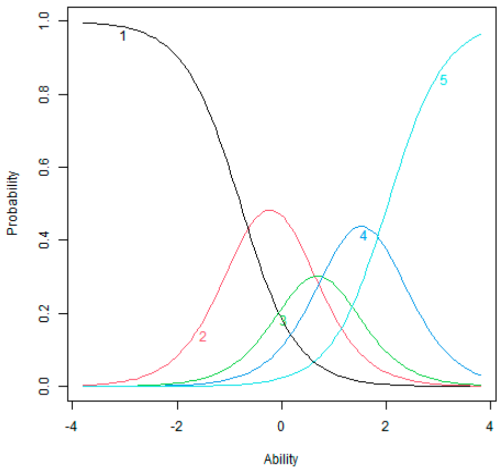

Figure S38. Item response category characteristic for item_6

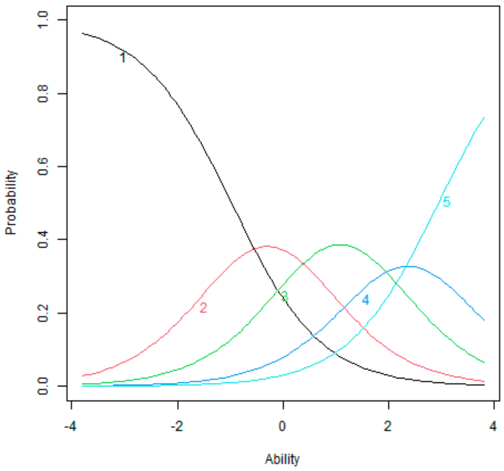

Figure S39. Item response category characteristic for item_13

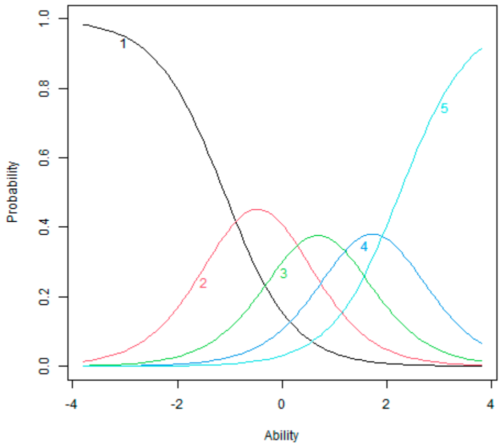

Figure S40. Item response category characteristic for item_20

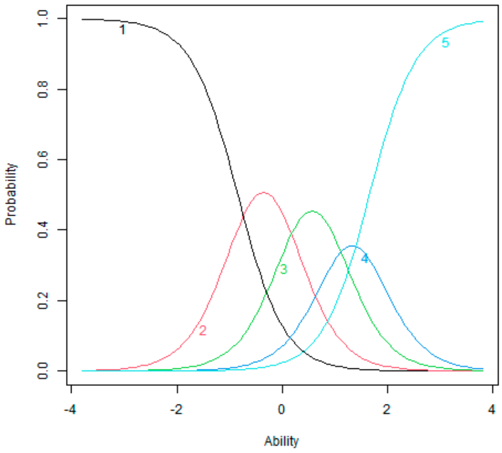

Figure S41. Item response category characteristic for item_27

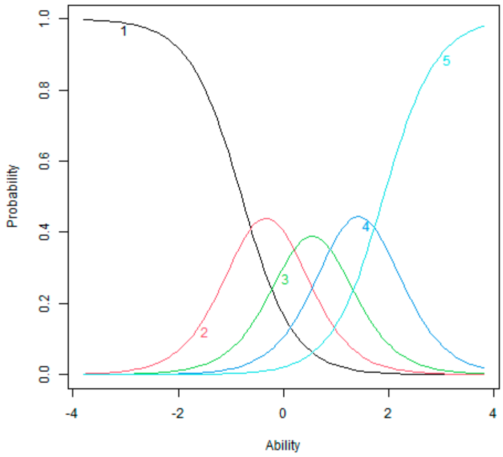

Figure S42. Item response category characteristic for item_34

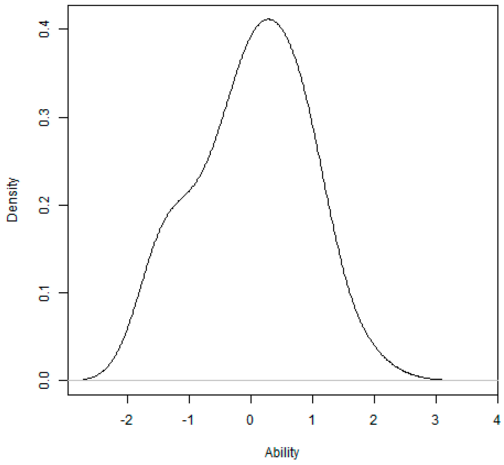

Figure S43. Kernel Density estimation for ability estimates (egocentrism)

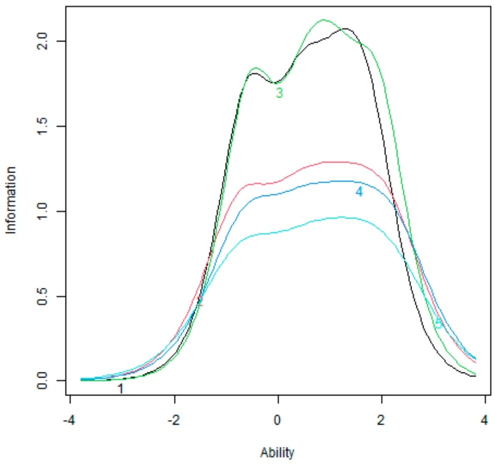

Figure S44. Item information curves for the egocentrism dimension

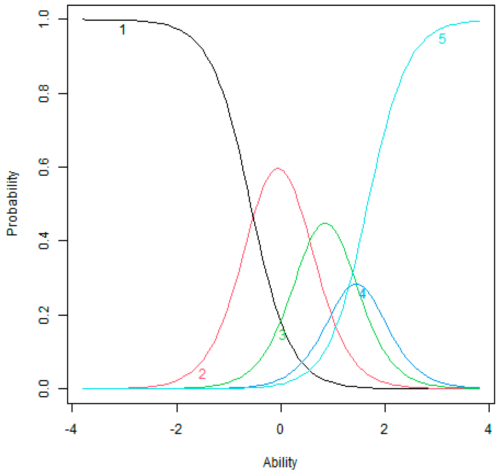

Figure S45. Item response category characteristic for item_7

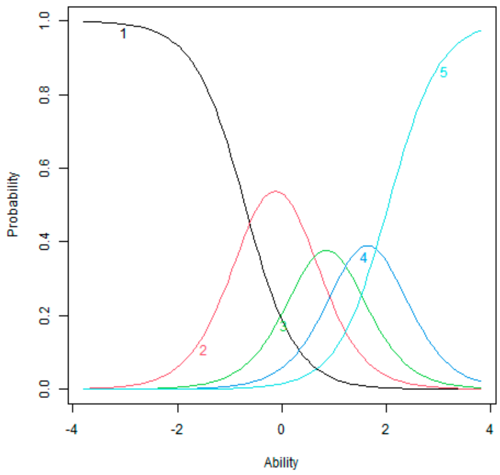

Figure S46. Item response category characteristic for item_14

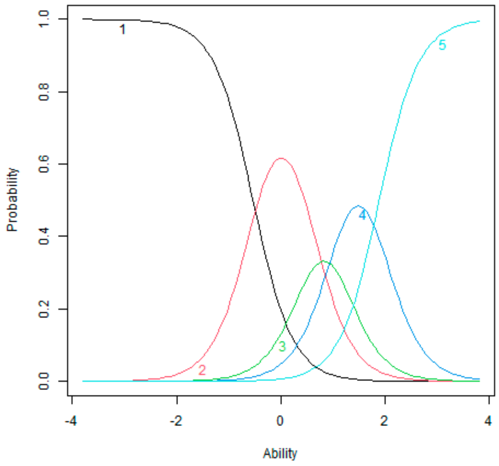

Figure S47. Item response category characteristic for item_21

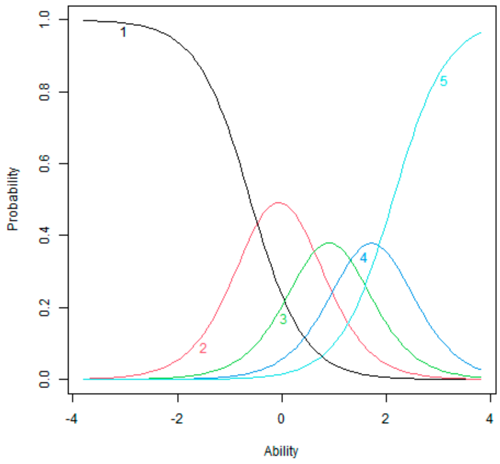

Figure S48. Item response category characteristic for item_28

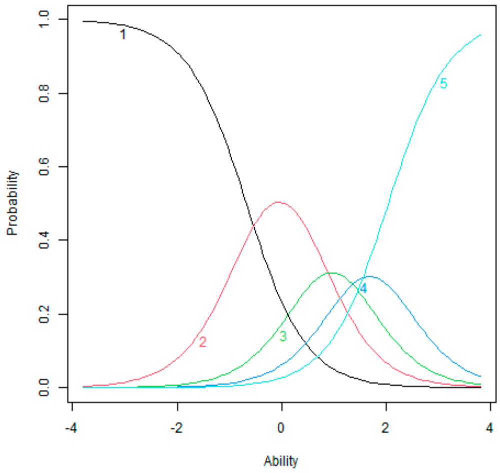

Figure S49. Item response category characteristic for item_35
